# Microbiota-derived metabolites and cardiovascular implications in Inflammatory Bowel Disease (IBD)

**DOI:** 10.1186/s10020-026-01493-6

**Published:** 2026-04-29

**Authors:** Ivna Olić, Nikola Pavlović, Marko Kumrić, Roko Šantić, Andre Bratanić, Joško Božić

**Affiliations:** 1 Department of Gastroenterology, University Hospital of Split, Split, 21000 Croatia; 2 Department of Pathophysiology, School of Medicine, University of Split, Split, 21000 Croatia; 3 Laboratory for Cardiometabolic Research, School of Medicine, University of Split, Split, 21000 Croatia; 4 Department of Internal Medicine, School of Medicine, University of Split, Split, 21000 Croatia

**Keywords:** Inflammatory bowel disease, Gut microbiota, Microbial metabolites, Cardioprotection, Cardiovascular disease, Dysbiosis

## Abstract

**Background:**

Inflammatory bowel disease (IBD), encompassing Crohn's disease and ulcerative colitis, is a chronic relapsing systemic disorder associated with significant extraintestinal manifestations. Emerging evidence indicates that cardiovascular disease represents a clinically important comorbidity in IBD, driven by persistent low-grade inflammation, endothelial dysfunction, and metabolic disturbances.

**Main body:**

The gut microbiota, recognized as a key regulator of host metabolism and immune homeostasis, contributes to cardiovascular physiology through the production of bioactive metabolites. These microbiota-derived metabolites — including short-chain fatty acids, secondary bile acids, trimethylamine N-oxide, tryptophan-derived indoles, and polyphenol-derived compounds — can exert cardioprotective or cardiotoxic effects depending on their balance and bioavailability. In IBD, intestinal dysbiosis and impaired epithelial barrier integrity profoundly alter microbial metabolic output, thereby disrupting systemic inflammatory and cardiovascular pathways. This review summarizes current evidence on the role of microbiota-derived metabolites in cardioprotection and examines how IBD-associated dysbiosis disrupts these protective mechanisms.

**Conclusion:**

Understanding the interplay between gut dysbiosis and cardiovascular risk in IBD may open new therapeutic avenues. Targeting the gut microbiota and its metabolic output represents a promising strategy to mitigate cardiovascular risk in this patient population.

## Introduction

Inflammatory Bowel Disease (IBD) refers to a group of chronic inflammatory conditions, principally Crohn’s disease (CD) and ulcerative colitis (UC). It is a relapsing disorder of the gastrointestinal system, characterized by recurrent cycles of disease activity and remission, driven by immune-mediated mechanisms. Despite advances in therapy, IBD has no curative treatment and can involve both intestinal and extraintestinal manifestations (Liang et al. 2024; Federici et al. 2023).

This inflammatory process gives rise to a broad spectrum of clinical manifestations, including abdominal discomfort, bloody diarrhea (hematochezia), fecal urgency, fatigue, unintended weight loss, nutritional deficiencies, fever, and musculoskeletal pain (Malik and Aurelio 2023). The global incidence and prevalence of IBD have risen substantially over recent decades, representing a growing public health challenge, especially in industrialized countries (Kaplan and Windsor 2021). The pathogenesis of IBD involves a complex interplay between the intestinal microbiota and the host immune system, shaped by genetic susceptibility and environmental influences (Rahaman et al. 2025; Pang et al. 2021). Patients with IBD can be stratified according to distinct alterations in fecal, urinary, and serum metabolomic profiles, revealing novel mechanisms and previously unrecognized disease associations (Bringer et al. 2022).

Cardiovascular diseases (CVDs) represent the leading cause of mortality and a major contributor to disability, arising largely from an interplay of socioeconomic, metabolic, behavioral, and environmental risk factors (Murray 2022). Atherosclerotic manifestations, particularly myocardial infarction (MI) and ischemic stroke, account for the majority of cardiovascular-related deaths globally (Tsao et al. 2022).

Previous studies have observed an increased risk of CVD in patients with IBD (Chen et al. 2024; Sun et al. 2024; Eriksson et al. 2024), while others suggested null associations (Dregan et al. 2014; Prasada et al. 2020). IBD has been implicated in the acceleration of atherosclerotic processes and the promotion of endothelial dysfunction, thereby contributing to an elevated risk of ischemic heart disease (Chen et al. 2022; Geng et al. 2026). Nevertheless, the precise relationship between IBD and CVD remains insufficiently elucidated. Although the increased risks of heart failure were shown across IBD, some studies estimated the relative risk were lower in UC (Sun et al. 2023; Aniwan et al. 2018). Existing evidence is inconsistent, with ongoing debate as to whether IBD constitutes an independent cardiovascular risk factor or primarily exerts its effects through interaction with established risk determinants. Notably, the observed strong association between IBD and delayed post-myocardial infarction pericarditis (Dressler’s syndrome) represents a clinically relevant and potentially novel insight (Sudhakaran et al. 2026).

Recent evidence from large population-based studies indicates an increased incidence of acute arterial events in patients with IBD, particularly among younger subjects and during phases of active disease (Alayo et al. 2023), and these findings are in concordance with the published results by Lee et al. (Lee et al. 2021a), who showed a higher prevalence of IBD among those with premature and extremely premature (age, < 40 y) atherosclerotic disease, but it remains unclear why younger individuals exhibit an increased CVD risk. Without a clearly defined mechanism, a multicenter cohort study also demonstrated a higher risk of cardiovascular disease in younger participants with IBD in comparison with older age groups, which may translate to a risk for premature atherosclerotic disease (Kirchgesner et al. 2018).

The gut microbiota constitutes one of the most complex ecosystems known, harboring dense microbial communities within the intestine and colon, with estimated concentrations of approximately 10^11^–10^12^ microorganisms per gram of intestinal contents (Lin and Medeiros 2023). The human gut microbiome is largely established during the first three years of life, during which progressive colonization by diverse microbial taxa is critical for the maturation and functional education of the developing immune system (Di Ciaula et al. 2023; Puljiz et al. 2023). In the adult human gut, the microbiome is predominantly composed of the bacterial phyla *Firmicutes* (*Bacillota*), *Bacteroidetes* (*Bacteroidota*), *Actinobacteriota*, *Proteobacteria* (*Pseudomonata*), and *Verrucomicrobiota* (Hul et al. 2024). Alterations in the composition and function of the gut microbiome, commonly referred to as dysbiosis, modulates disease development through the production of microbial metabolites, as well as by influencing transcription factor activity, intestinal barrier integrity, immune tolerance, cytokine production, and the circadian regulation of host tissues (Martin-Pelaez et al. 2020; Cai et al. 2022a). Gut dysbiosis is increasingly recognized as a critical factor in the development of several major CVD manifestations, including hypertension, atherosclerosis, and heart failure (Rajendiran et al. 2021).

Given that IBD is characterized by profound alterations in gut microbiota composition and function, microbiota-derived metabolites represent a plausible mechanistic link between intestinal inflammation and cardiovascular pathology. This review focuses on the role of these metabolites in cardiovascular outcomes and examines how their dysregulation in IBD may contribute to cardiovascular risk.

**Fig. 1:**
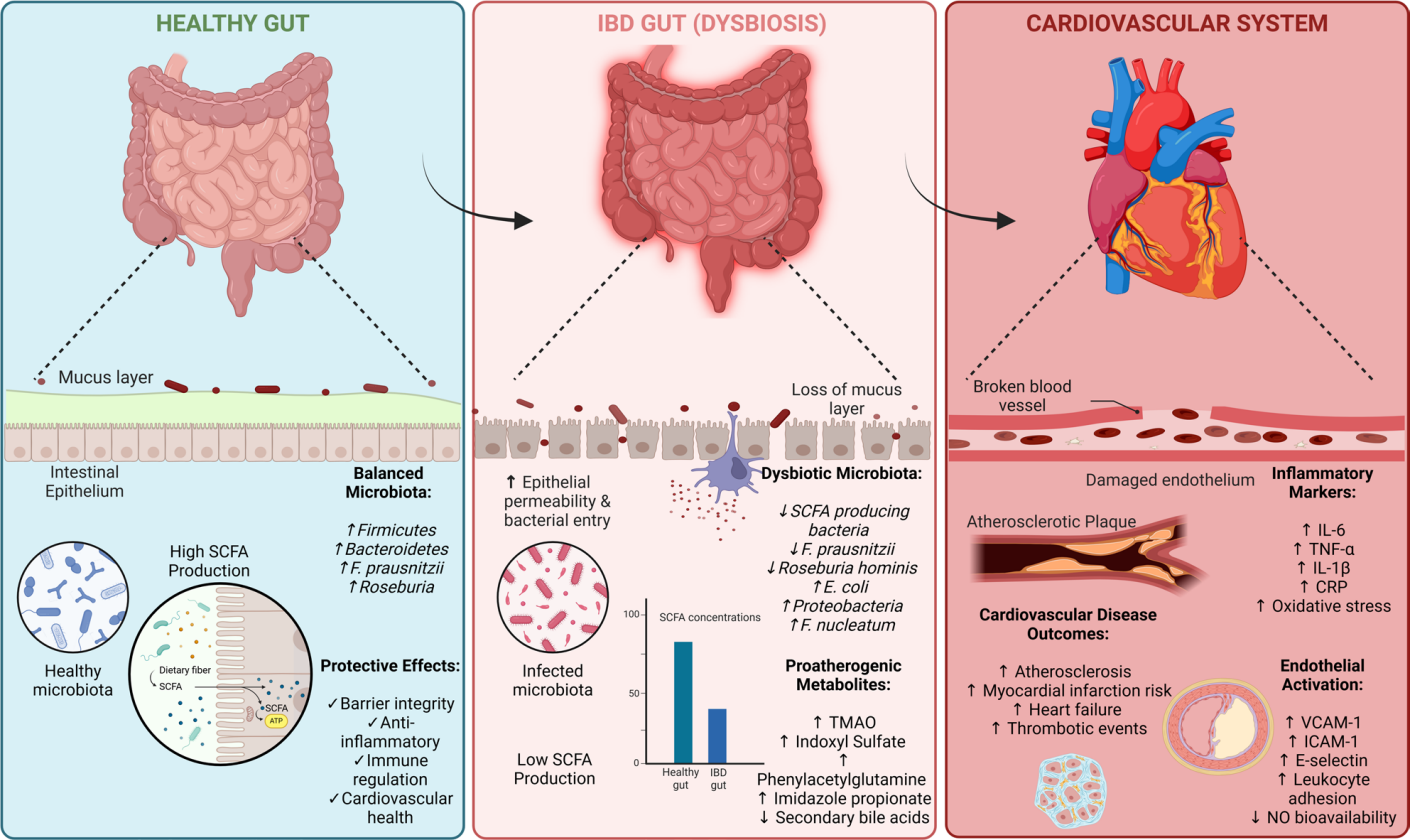
Gut-Cardiovascular Axis in Health and IBD. In a healthy gut, the integrity of the mucus layer and intestinal epithelium preserves barrier function and restricts bacterial translocation. A balanced microbiota, enriched with short-chain fatty acids (SCFA)-producing bacteria such as Firmicutes, Bacteroidetes, Faecalibacterium prausnitzii, and Roseburia species, facilitates robust SCFA production from dietary fiber. SCFAs support epithelial energy metabolism, mediate anti-inflammatory signaling, regulate immune responses, and confer cardiovascular protection. In IBD, the dysbiotic microbiota exhibits a reduced abundance of SCFA-producing taxa, such as Faecalibacterium prausnitzii and Roseburia hominis, alongside an expansion of potentially pathogenic or pro-inflammatory bacteria, including Escherichia coli, Proteobacteria, and Fusobacterium nucleatum. Simultaneously, microbial metabolism favors the generation of pro-atherogenic metabolites, such as trimethylamine N-oxide (TMAO), indoxyl sulfate (IS), phenylacetylglutamine (PAGIn), and imidazole propionate, as well as altered bile acid profiles. Gut-derived inflammatory signals and metabolites enter the systemic circulation and contribute to endothelial dysfunction. This dysfunction is characterized by endothelial activation, as indicated by increased vascular cell adhesion molecule-1(VCAM-1), intercellular adhesion molecule-1(ICAM-1), and E-selectin; reduced nitric oxide (NO) bioavailability; heightened oxidative stress; and elevated inflammatory markers, including interleukin-6 (IL-6), tumor necrosis factor-alpha (TNF-α), interleukin-1 beta (IL-1β), and C-reactive protein (CRP). These processes promote vascular injury, atherosclerotic plaque development, and an increased risk of adverse cardiovascular outcomes, such as atherosclerosis, MI, heart failure, and thrombotic events. Created with BioRender

Figure 1 illustrates the proposed interplay between IBD, gut microbiota dysbiosis, microbiota-derived metabolites, systemic inflammation, and cardiovascular disease.

## Gut microbiota dysbiosis in IBD: microbiota-derived metabolites as systemic mediators

Recent studies have increasingly demonstrated that the gut microbiota and its metabolites play a critical role in the development of both cardiovascular and non-cardiovascular diseases. This process is driven by persistent low-grade inflammation associated with intestinal barrier impairment, which allows microbial products to enter the systemic circulation. Moreover, gut dysbiosis contributes to the generation of pro-inflammatory metabolites, further amplifying inflammatory signaling in a microbiota-dependent manner (Nesci et al. 2023; Campbell et al. 2023).

Functional dysbiosis in patients with IBD is characterized by an increased ratio of facultative to obligate anaerobes, a depletion of SCFA-producing species, overrepresentation of facultative anaerobes such as adherent-invasive *Escherichia coli* and various Proteobacteria species, and reduced abundance of beneficial *Firmicutes*, particularly *Faecalibacterium prausnitzii* and *Roseburia hominis* (Lloyd-Price et al. 2019; Scanu et al. 2024).

In addition to dysbiosis, patients with IBD exhibit intestinal barrier dysfunction arising from multiple interrelated mechanisms, including reduced mucus layer integrity, altered expression of tight junction proteins, impaired production of antimicrobial peptides, and increased epithelial cell apoptosis. This disruption of the epithelial barrier facilitates the translocation of bacterial antigens into the systemic circulation. Specifically, the entry of microbial product lipopolysaccharide (LPS) into the bloodstream induces low-grade endotoxemia, which has been associated with prothrombotic signaling pathways, including toll-like receptor 4 (TLR4)-mediated inflammatory activation. Low-grade endotoxemia, defined as persistently elevated circulating levels of bacterial LPS > 20 ng/mL originating from Gram-negative microorganisms (Jian et al. 2025; Carnevale et al. 2020; Cani et al. 2007), has been documented in patients with IBD. Evidence from several clinical studies indicates that detectable endotoxemia occurrs more frequently in CD than in UC. Across these investigations, circulating LPS levels have been shown to correlate positively with indices of disease activity and clinical severity, supporting a link between systemic endotoxin exposure and the inflammatory burden in both disease phenotypes (Fusco et al. 2025; Violi et al. 2023; Candelli et al. 2021).

Biological sex and ethnicity are important determinants of gut microbial profiles, which, in turn, modulate the clinical course of IBD and influence cardiovascular risk via the gut-heart axis. In women, estrogen has been shown to favor a more diverse intestinal microbiome by stimulating the proliferation of health-associated taxa, including *Akkermansia muciniphila* and members of the *Clostridia* class (Kim et al. 2020; Valeri and Endres 2021). This hormonal effect is associated with an increased *Firmicutes*-to-*Bacteroidetes* ratio; however, despite its widespread use as a descriptive marker of gut microbial composition, the reliability and biological relevance of this ratio have been increasingly questioned. Such alterations in microbiota have been linked to cardiovascular protection and may contribute to reduced susceptibility to atherosclerotic disease, particularly in premenopausal females (Peters et al. 2024). In a comparable manner, individuals of African American ancestry frequently exhibit reduced gut microbial diversity alongside an increased relative abundance of potentially pathogenic taxa, including *Fusobacteriota* and *Bacteroides*, when compared with white populations (Piawah et al. 2023; Borrello et al. 2022). These differences are largely attributed to dietary patterns and broader social and environmental determinants of health, and are associated with a greater susceptibility to severe IBD manifestations, such as perianal disease, abscess formation, and anal fissures, particularly in patients with CD (Borum 2023).

Archaea are unicellular microorganisms phylogenetically distinct from bacteria. In the gut, methanogens predominate, utilizing hydrogen and carbon dioxide to produce methane and thereby supporting efficient microbial fermentation (Houshyar et al. 2021; Koskinen et al. 2017). Insufficient methanogenic activity may lead to hydrogen accumulation, reduced SCFA production, and a shift toward less favorable metabolic pathways (Quiroga-Centeno et al. 2025). However, not all archaea exert beneficial effects; *Methanosphaera stadtmanae* is enriched in IBD and has been shown to strongly activate dendritic cells, inducing the release of pro-inflammatory cytokines such as TNF-α, IL-6, and IL-1β (Ruden 2025; Bang et al. 2014). Evidence from Scanlan et al. demonstrated a marked reduction in *Methanobrevibacter smithii* in IBD patients (Scanlan et al. 2008). More recently, Cisek et al. suggested that methanogenic archaea may modulate IBD susceptibility in both pediatric and adult populations. Notably, total methanogen abundance was significantly lower in both the UC and CD cohorts compared with healthy subjects (Cisek et al. 2024; Just et al. 2013). Certain archaeal species produce bioactive compounds, of particular relevance, the archaeal halocin H6 has demonstrated cardioprotective effects by modulating Na⁺/H⁺ exchanger (NHE) activity, thereby contributing to blood pressure regulation and the prevention of myocardial injury (Lee et al. 2013). NHE is a major mechanism for intracellular pH regulation in cardiomyocytes, when cytoplasm acidification is induced. Ischemia conditions induce sustained intracellular acidification that strongly activates NHE, leading initially to detrimental fast restoration of pH followed by intracellular Na + and Ca + 2 overflow. NHE inhibition avoids Na + and Ca + 2 overload into cell, thus preventing myocardium damage (Lequerica et al. 2006).

Archae *Methanobrevibacter smithii* colonization significantly lowers plasma TMAO levels utilizing trimethylamine (TMA) and shows a trend toward reduced atherosclerotic burden in Apoe⁻/⁻ mice (Ramezani et al. 2018). Microbes capable of metabolizing TMA prior to its absorption may therefore represent a promising therapeutic approach for limiting TMAO-associated cardiovascular risk.

The gut microbiota converts cholesterol into coprostanol, a non-absorbable sterol excreted in feces. The conversion of cholesterol to coprostanol is impaired in IBD (Matysik et al. 2025). This conversion is clinically relevant, as it is associated with reduced circulating cholesterol levels (Kazemian et al. 2020). The abundance of cholesterol-metabolizing bacteria has been shown to correlate closely with factors influencing cholesterol conversion. Intestinal microbes capable of converting cholesterol to coprostanol include *Eubacterium coprostanoligenes*, *Bacteroides* spp., *Lactobacillus* spp., and *Bifidobacterium* spp.(Kenny et al. 2020). Hence, research on coprostanol-producing bacterial strains may have clinical potential to modulate the gut microbiota and reduce cardiovascular risk (Shoarishoar et al. 2024; Kriaa et al. 2019), but further investigation is required, as the underlying mechanisms remain incompletely understood.

*Fusobacterium nucleatum* is increasingly detected in intestinal biopsies from patients with IBD (Wang et al. 2024a). A key virulence factor of *F. nucleatum* is the ability to adhere and invade endothelial cells via the FadA adhesin which activates TLR2/4-mediated nuclear factor kappa-light-chain enhancer of B cells (NF-κB) and mitogen-activated protein kinase (MAPK) signaling (Majumder et al. 2024). This activation promotes the expression of IL-6, TNF-α, and IL-1β, as well as endothelial adhesion molecules, facilitating leukocyte adhesion and transmigration (Curia et al. 2022). These events leads to subsequent atheroma formation. Additionally, *F. nucleatum*-derived lipopolysaccharides enhancing oxidative stress by activating nicotinamide adenine dinucleotide phosphate (NADPH) oxidase to produce reactive oxygen species (ROS). ROS-driven oxidation of low-density lipoprotein (oxLDL) accelerates foam cell formation and destabilizes pre-existing plaques. Collectively, these molecular mechanisms establish a pathophysiological connection between chronic periodontal colonization and systemic atherosclerotic progression (Wang et al. 2024b). Furthermore, periodontal microbes may contribute to intestinal inflammation in IBD, reinforcing the concept of a shared pathogenic axis between oral and gut inflammatory diseases (Ozmeric et al. 2018).* F. nucleatum* also drives foam cell formation by increasing macrophage lipid uptake and reducing cholesterol efflux, contributing to atherosclerotic lesion development (Chen et al. 2023).

## Proatherogenic metabolites and mechanisms

### Trimethylamine N-oxide (TMAO)

TMAO is a metabolite generated by gut microbiota from dietary precursors such as choline, L-carnitine, and betaine, which are abundant in foods like red meat, eggs, and dairy. 2023(Agus et al. 2021; Banno et al. 2023 Benson et al. 2023). TMA is then absorbed into the circulation and transported to the liver, where primarily flavin-containig monooxygenase 3 (FMO3) oxidize it into TMAO. Emerging evidence identifies TMAO as both a marker of host-microbiota metabolic interactions and an active mediator of immune-inflammatory pathways linking gut dysbiosis to intestinal mucosal injury (Maki et al. 2024). TMAO contributes to IBD pathogenesis by modulating key signaling pathways, including protein kinase RNA-like endoplasmic reticulum kinase (PERK)-mediated endoplasmic reticulum stress and NF-κB-dependent cytokine production. TMAO activates NF-κB in aortic endothelial cells via mitogen-activated protein kinase (MAPK)/extracellular regulated protein kinases (ERK) signaling, promoting leukocyte adhesion and vascular inflammation (Wang et al. 2025; Geng et al. 2018). Accordingly, NF-κB appears to function as a key convergence node through which TMAO-induced MAPK/ERK signaling is translated into endothelial activation, while parallel MAPK/JNK-dependent CD36 upregulation promotes foam-cell formation and progression of atherosclerosis (Wang et al. 2025; Geng et al. 2018; Seldin et al. 2016). TMAO promotes atherogenesis through multiple pathways. In ApoE⁻/⁻ mice fed a high-fat diet, TMAO supplementation enhances plaque development, macrophage infiltration, and CD36 and pro-inflammatory cytokine expression. In vitro, inhibition of the MAPK/c-Jun n-terminal kinase (JNK) pathway attenuates TMAO-induced CD36 upregulation and foam cell formation (Seldin et al. 2016). Once formed, TMAO enters the bloodstream (Yoo et al. 2022) and is eventually excreted in urine (Cho et al. 2017). Renal function represents a major determinant of circulating TMAO concentrations and may confound associations between TMAO and cardiovascular outcomes. Consequently, alterations in gut microbial composition or impaired renal function can lead to elevated circulating TMAO levels (Coutinho-Wolino et al. 2021). High TMAO concentrations have been associated with an increased risk of CVD (Kim et al. 2025; Budoff et al. 2025), chronic kidney disease (CKD) (Lee et al. 2021b), and, to a lesser extent, IBD, although causal relationships remain to be fully elucidated. Mechanistically, TMAO contributes to disease pathogenesis by modulating inflammatory signaling, oxidative stress, and fibrotic pathways, and by influencing gut microbial homeostasis (Petersen and Round 2014). TMAO is being explored as a potential adjunctive biomarker for cardiovascular risk stratification in patients with IBD, although its incremental predictive value beyond classical risk models remains uncertain (Kim et al. 2025; Wilson et al. 2015).

### Indoxyl sulfate and tryptophan metabolites

Indoxyl sulfate (IS) is a microbiota-derived tryptophan metabolite that accumulates systemically as renal clearance deteriorates, owing to reduced tubular secretion and glomerular filtration, making it a prominent uremic toxin in CKD (Novokhodko et al. 2026). IS also promotes endothelial dysfunction by inducing oxidative stress and inflammatory signaling. IS enhances NADPH oxidase-dependent reactive oxygen species generation, reduces nitric oxide bioavailability, and activates the aryl hydrocarbon receptor, leading to NF-κB-mediated endothelial activation, increased expression of adhesion molecules, and leukocyte recruitment. In addition, IS impairs endothelial progenitor cell function, limiting vascular repair capacity. Clinically, higher circulating IS levels are associated with reduced flow-mediated dilation and increased vascular stiffness, linking IS accumulation to elevated cardiovascular risk (Harlacher et al. 2022).

Elevated circulating IS concentrations, in parallel with a higher prevalence of MI among individuals with increased fecal calprotectin, are consistent with a clinically meaningful gut-heart axis, suggesting that intestinal inflammatory burden may be linked to adverse cardiovascular outcomes (Yamamoto et al. 2021). In fact, children with CD, when compared with age-matched UC patients, exhibit increased urinary excretion of indoxyl sulfate and its oxidized derivative, hydroxyindoxyl sulfate, alongside reduced kynurenine levels (Heinzel et al. 2024).

### Phenylacetylglutamine (PAGln)

PAGln is a gut microbiota-derived metabolite formed from dietary phenylalanine. Gut microbes first convert phenylalanine into phenylacetic acid, which is subsequently conjugated with glutamine in hepatocytes and renal cells to generate PAGln (Shokravi et al. 2025). Circulating PAGln levels are increased in subjects with CD compared with healthy individuals (Feng et al. 2023). In treatment-naïve CD, dysbiosis promotes expansion of PAGln-producing Proteobacteria, contributing to higher systemic PAGln concentrations (Feng et al. 2023). Beyond its intestinal effects, PAGln exerts cardiovascular actions by modulating host adrenergic signaling. It acts as a negative allosteric modulator of β2-adrenergic receptors in murine and human ventricular cardiomyocytes, reducing cardiac contractility (Saha et al. 2024). Also, the administration of the murine analog phenylacetylglycine (PAGly) aggravates colitis, whereas human ex vivo findings indicate that PAGln augments platelet reactivity and induces expression of prothrombotic genes (Feng et al. 2023). Clinically, increased plasma PAGln is independently connected with elevated risk of hospitalization and mortality in individuals with heart failure (Tang et al. 2024). Mechanistically, by enhancing intracellular Ca^2^⁺ cycling, PAGIn activates calcium/calmodulin-dependent protein kinase II (CaMKII) which leads aberrant phosphorylation of ion-handling proteins, driving to calcium leak from the sarcoplasmic reticulum, delayed afterdepolarizations, and increased susceptibility to ventricular and atrial arrhythmias. Concurrently, PAGln activates TLR4, triggering the AKT-mTOR signaling pathway, which enhances inflammatory gene expression, promotes cardiomyocyte hypertrophy and fibroblast activation, and ultimately contributes to myocardial fibrosis, adverse remodeling, and progressive cardiac dysfunction (Zhang et al. 2022; Vaez et al. 2023; Fu et al. 2024; Nageswaran et al. 2025).

### Imidazole propionate

Imidazole propionate (ImP), a histidine-derived metabolite, impairs endothelial function and promotes vascular inflammation. Mechanistically, it disrupts phosphoinositide 3-kinase (PI3K)/AKT signaling, leading to activation of the transcription factor forkhead box protein O1 (FOXO1) and consequent inhibition of endothelial cell proliferation and migration. This dysfunction compromises endothelial repair after vascular injury and accelerates atherosclerosis, linking ImP to the pathogenesis of atherosclerotic cardiovascular disease (Puca et al. 2025). Accordingly, ImP is increased in individuals with heart failure and is a predictor of overall survival (Verdugo-Meza et al. 2020).

## Cardioprotective microbial metabolites

### Indole-3-propionic acid (IPA)

In IBD, gut dysbiosis disrupts bacterial tryptophan metabolism, leading to reduced production of beneficial indole derivatives. One such metabolite, IPA, is markedly lower in individuals with active IBD compared to healthy subjects (Menni et al. 2019). Elevated IPA levels have been inversely correlated with arterial stiffness, insulin resistance, fasting glucose, and visceral adiposity (Ye et al. 2022). Deficiency of IPA deprives the host of an important immunoregulatory signal, potentially amplifying systemic inflammation (Ye et al. 2022). Therefore, chronic IPA depletion resulting from persistent dysbiosis in IBD may contribute to metabolic disturbances and increased inflammatory signaling, thereby creating a systemic milieu conducive to adverse cardiac remodeling. However, while experimental and observational data support an association between reduced IPA levels and increased cardiovascular risk, the direct mechanistic role of IPA deficiency in driving cardiac remodeling and heart failure progression remains incompletely elucidated.

### Short-Chain Fatty Acids (SCFAs)

SCFAs, such as butyrate, acetate, and propionate, generated in the colon by commensal microbes, including *Faecalibacterium prausnitzii*, *Roseburia*, and members of the Ruminococcaceae family, through the fermentation of dietary fibers and resistant starch, play a key role in maintaining intestinal homeostasis by supporting the growth of beneficial microbes, modulating immune responses, and strengthening gut barrier function (Mills et al. 2019; Nishida et al. 2018). In healthy individuals, SCFAs further promote regulatory T cell activity, suppress pro-inflammatory mediators, and increase colonic epithelial oxygen consumption, hence contributing substantially to energy metabolism, immune homeostasis, and the maintenance of epithelial barrier integrity. In IBD, however, SCFA production is markedly impaired, exacerbating disease progression (Mills et al. 2019; Nishida et al. 2018; Wang et al. 2026; Parada Venegas et al. 2019).

Firstly, reduced SCFAs in IBD patients impair intestinal barrier integrity, facilitating translocation of microbial products such as LPS into the circulation. This promotes systemic inflammation and increases circulating pro-inflammatory cytokines (e.g., IL-6, TNF-α) (Wang et al. 2026; Parada Venegas et al. 2019).

Butyrate, the main energy source for colonocytes, supports mitochondrial respiration and epithelial health. In UC, diminished butyrate utilization correlates with decreased expression of transporters, and key metabolic enzymes, including acetyl-CoenzymeA synthetase, resulting in energy deficits and compromised barrier function (Zhang et al. 2025a). SCFAs also inhibit NF-κB signaling, lowering the production of pro-inflammatory cytokines (e.g., IL-1β, TNF-α) in immune cells (Hao et al. 2021). Moreover, they promote the differentiation of regulatory T cells (Tregs) through aryl hydrocarbon receptor (AhR) and peroxisime proliferator-activated receptor (PPAR)-γ signaling, balancing Th17/Treg immune responses. Butyrate specifically enhances Treg development by upregulating FOXP3 expression (Hays et al. 2024). SCFAs further strengthen the intestinal barrier by increasing tight junction proteins (ZO-1, occludin) and mucin (MUC2) production, while butyrate enhances Slc26a3 expression by inhibiting the histone deacetylase 8 (HDAC8)/NF-κB pathway, reinforcing epithelial integrity (Peng et al. 2024; Luu et al. 2019).

Pentanoate, another SCFA, exhibits strong HDAC inhibition but does not stimulate mucosal Treg expansion. Its anti-inflammatory activity manifests through increased IL-10 production in B regulatory cells and CD4 + effector T cells, alongside suppression of pathogenic Th17 cells. Mechanistic studies reveal that IL-10 induction by pentanoate relies on enhanced mTOR signaling, increased glucose-derived pyruvate oxidation, and elevated acetyl-CoA generation (Parada Venegas et al. 2019; Zhou et al. 2018).

In IBD mucosa, depletion of SCFA-producing bacteria (e.g., *F. prausnitzii*, Ruminococcaceae) reduces SCFA availability. *F. prausnitzii* abundance correlates positively with colonic butyrate levels and inversely with disease activity. Even when SCFAs are present, epithelial cells in IBD exhibit impaired uptake and oxidation of butyrate due to mitochondrial dysfunction and inflammation-driven gene repression (Wang et al. 2024c).

But, recent evidence suggests that higher SCFA concentrations do not universally confer benefits. Under inflammatory conditions, the effects of SCFAs appear to be context-dependent, varying with concentration, receptor expression, disease state, and the host metabolic milieu.

Butyrate and propionate, in the presence of TLR agonists, activate the NOD-like receptor protein (NLRP3) inflammasome through a caspase-8-dependent mechanism. This occurs via HDAC inhibition, which suppresses transcription of FLICE-like inhibitory protein (cFLIP) and IL-10. These findings indicate that SCFA signaling is highly context-dependent, because under inflammatory TLR stimulation it may promote caspase-8-dependent NLRP3 activation and IL-1β release rather than exert exclusively anti-inflammatory effects (Hao et al. 2021; Collins et al. 2023). Notably, SCFA-driven NLRP3 activation occurs independently of potassium efflux and does not induce cell death; instead, it triggers hyperactivation of immune cells and increased IL-1β secretion (Collins et al. 2023).

Importantly, while SCFAs themselves are generally anti-inflammatory, in IBD patients their deficiency—rather than excess—is the key issue. Thus, the cardiovascular impact is largely indirect, mediated through loss of protective mechanisms rather than active promotion of inflammation by SCFAs.

### Bile Acids (BAs)

BAs, produced by the liver and subsequently modified by gut microbiota, play essential roles in lipid absorption and immune homeostasis. Commensal bacteria, including *Bacteroides*, *Clostridia*, and *Eubacterium*, transform primary BAs such as cholate and chenodeoxycholate into secondary BAs, including deoxycholate and lithocholate, via 7α-dehydroxylation. In healthy individuals, secondary BAs activate the farnesoid X receptor (FXR) and the G-protein-coupled bile acid receptor TGR5, eliciting anti-inflammatory effects and strengthening epithelial barrier function. In IBD, diminished 7α-dehydroxylase activity in the gut microbiota reduces secondary BA production, impairing FXR/TGR5 signaling and contributing to intestinal inflammation and barrier dysfunction (Cai et al. 2022b).

Secondary BAs, such as deoxycholate, stimulate FXR signaling in intestinal epithelial cells (IECs), leading to increased expression of tight junction proteins and antimicrobial peptides, thereby reinforcing barrier integrity. In UC, impaired FXR activation compromises epithelial restitution and facilitates pathogen adherence (Zheng et al. 2021).

Activation of TGR5 by BAs promotes secretion of glucagon-like peptide-1 (GLP-1) and drives macrophage polarization toward an anti-inflammatory M2 phenotype. In IBD, reduced TGR5 signaling contributes to enhanced Th1/Th17 responses and heightened mucosal inflammation (Wang et al. 2018). Taken together, impaired microbiota-dependent FXR/TGR5 signaling may represent a plausible mechanistic bridge between IBD and cardiovascular risk, because reduced secondary bile acid signaling compromises epithelial restitution, amplifies Th1/Th17-skewed mucosal inflammation, and favors pro-atherogenic metabolic and macrophage responses (Cai et al. 2022b; Zheng et al. 2021; Wang et al. 2018; Pols et al. 2011; Zhang et al. 2023).

Dysregulation of FXR and TGR5 signaling can negatively affect cardiac function by promoting atherosclerosis and disturbances in myocardial metabolism, collectively contributing to cardiac dysfunction (Pols et al. 2011; Zhang et al. 2023). In chronic heart failure, intestinal hypoperfusion disrupt enterohepatic bile acid circulation, resulting in an increase in circulating secondary BAs and a decrease in primary BAs. A higher secondary-to-primary BA ratio has been linked to worse clinical outcomes, including elevated mortality. Although IBD and heart failure exhibit contrasting BA profiles, primary BAs predominate in IBD, whereas secondary BAs are elevated in heart failure, both conditions converge on impaired BA receptor signaling, triggering downstream inflammatory and fibrotic pathways. Interpretation of BAs profiles requires disease-specific context, as similar alterations may have divergent implications in IBD and heart failure. The precise effects of IBD-associated BA alterations on cardiac function remain unclear, and further research is needed to clarify the role of BA dysregulation in IBD-related heart failure (Janeiro et al. 2018).

In Table 1 we present the principal mechanisms and pathways of the gut-heart axis that are disrupted or accentuated both within IBD spectrum and cardiovascular system.

**Table 1:** Summary of main pathways included in gut-heart axis (Seldin et al. 2016; Harlacher et al. 2022; Zhang et al. 2022, 2023; Vaez et al. 2023; Fu et al. 2024; Nageswaran et al. 2025; Hays et al. 2024; Collins et al. 2023; Cai et al. 2022b; Pols et al. 2011)

Pathway	Microbiome related trigger	Cardiovascular effect	IBD impact	Level of evidence
NF-κB	TMAO, IS, LPS, dysbiosis, barrier disruption	Endothelial dysfunction, vascular inflammation, atherosclerosis	Central driver of mucosal inflammation and cytokine production	Strong (human + animal)
NLRP3 inflammasome	Microbial products, ROS	IL-1β/IL-10 release, plaque instability, myocardial injury	Promotes intestinal inflammation and epithelial damage	Strong (animal + human associative)
FXR	Microbiota-modified bile acids	Lipid regulation, anti-atherogenic effects	Impaired signaling contributes to inflammation and dysbiosis	Moderate-strong
TGR5	Secondary bile acids	Anti-inflammatory, improved endothelial and metabolic function	Reduced activity linked to enhanced intestinal inflammation	Moderate
PPAR-γ	SCFA, Fiber fermentation	Blood pressure regulation, anti-inflammatory, vascular protection	Reduced SCFAs impair barrier integrity and immune homeostasis	Moderate-strong
AKT-mTOR	PAGln, TLR4	promotes cardiomyocyte hypertrophy, fibroblast activation	Aggravates colitis	Strong (animal = human)

## Therapeutic perspectives

Targeting the gut microbiota and its metabolites represents a promising strategy to mitigate cardiovascular risk in IBD. Interventions include dietary modification (e.g., increased fiber intake), prebiotics and probiotics, microbial enzyme inhibitors (e.g., TMA lyase inhibitors), and fecal microbiota transplantation (FMT). These approaches aim to enhance cardioprotective metabolites (e.g., SCFAs) while reducing proatherogenic compounds (e.g., TMAO). Most current evidence supporting metabolite-targeted interventions in IBD-associated cardiovascular risk derives from preclinical studies or observational human data, with limited randomized controlled trials available.

Various interventions aimed at reducing circulating TMAO levels, such as 3,3-dimethyl-1-butanol (DMB), probiotics, antibiotics, resveratrol, and enalapril, have been explored for their potential to mitigate cardiovascular risk and slow the progression of another chronic disorder, CKD (Ianiro et al. 2016). Collectively referred to as TMAO-lowering therapies (TLT), these approaches represent a promising avenue for cardiometabolic protection.

At present, strategies to modulate gut microbiota with the specific goal of reducing TMAO remain largely conceptual, and robust clinical evidence is limited. Since TMAO is generated from its precursor TMA, therapeutic approaches that inhibit TMA formation in the gut could offer a targeted and mechanistically rational method to control systemic TMAO levels and reduce associated disease risks.

Antibiotic therapy may modulate the clinical course of IBD through multiple mechanisms, including a reduction in intraluminal bacterial load and qualitative changes in the intestinal microbiota. By limiting bacterial adherence, mucosal invasion, and translocation, antibiotics may attenuate host-microbe interactions that contribute to intestinal inflammation. These effects can be accompanied by alterations in microbial metabolic activity, potentially favoring increased SCFA production. Furthermore, suppression of bacterial enzymatic functions may be associated with subsequent clinical improvement (Gupta, et al. 2016; Zhang et al. 2024). Nevertheless, short-term antibiotic administration has not been shown to confer meaningful long-term therapeutic benefits in patients with CD, suggesting that the effectiveness of antibiotic therapy may vary across IBD subtypes. Importantly, the potential long-term impact of antibiotic exposure on the intestinal microbiome, along with the risk of promoting antibiotic-resistant organisms, constitutes a major concern and underscores the need for a judicious, balanced therapeutic approach (Zhang et al. 2024).

The findings of Spasova et al. (Spasova et al. 2024) demonstrate that supplementation with *Lactobacillus plantarum* GLP3 over 12 weeks effectively reduced circulating TMAO levels in patients with a history of CVD. Given the established association between elevated TMAO concentrations and increased cardiovascular risk, this reduction suggests a potential cardioprotective effect of the probiotic intervention. The results support the role of targeted modulation of the gut microbiota as a complementary strategy in cardiovascular risk management among very high-risk patient populations.

*Lactobacillus fermentum* MJM60397 exhibited appropriate safety characteristics and key probiotic properties, including survival under simulated gastrointestinal conditions, epithelial adherence, and bile salt hydrolase activity. In a diet-induced hypercholesterolemia mouse model, MJM60397 supplementation led to a marked reduction in hepatic total and low-density lipoprotein (LDL) cholesterol levels (Palaniyandi et al. 2020). This effect was associated with enhanced fecal bile acid excretion and increased hepatic LDL receptor expression. The findings indicate that the cholesterol-lowering action of MJM60397 is primarily mediated through disruption of enterohepatic bile acid circulation via bile acid deconjugation. Collectively, these results support the potential of *L. fermentum* MJM60397 as a probiotic candidate for cholesterol management (Palaniyandi et al. 2020).

*Lactobacillus plantarum* HNU082 significantly attenuated disease severity in a dextran sulfate sodium-induced mouse model of UC, as evidenced by improvements in clinical, inflammatory, and histopathological parameters (Wu et al. 2022). The probiotic exerted its effects by coordinating the restoration of intestinal mucosal barrier function, including modulation of gut microbiota composition, enhancement of SCFA production, and reinforcement of epithelial and mucus barriers. In addition, Lp082 suppressed mucosal inflammation by downregulating proinflammatory cytokines and inhibiting NF-κB signaling while promoting anti-inflammatory mediators. These findings support the therapeutic potential of Lp082 as a probiotic-based strategy for UC (Wu et al. 2022).

Prebiotics are nondigestible dietary components that selectively promote the proliferation and metabolic activity of beneficial intestinal microorganisms, thereby contributing to host health (Silva et al. 2021; Huang et al. 2021). Growing preclinical evidence indicates that prebiotic interventions may ameliorate metabolic disorders, including obesity and type 2 diabetes mellitus (Macfarlane and Macfarlane 2011), by fostering a gut microbial milieu that favorably modulates host metabolic pathways. In addition, prebiotics enhance the intestinal production of SCFAs (Roller et al. 2004). Accumulating data further suggest a protective role of prebiotics in CVD prevention through reductions in total and LDL cholesterol levels, as well as attenuation of chronic inflammation (Huang et al. 2021; Zhang et al. 2025b). Supporting this concept, recent preclinical studies have shown that arabinoxylan supplementation improves choline-induced intestinal barrier impairment by restoring tight junction protein expression, suppressing PERK signaling, and decreasing trimethylamine accumulation (Khan et al. 2019).

Polyphenolic compounds represent a diverse class of plant-derived secondary metabolites with well-documented anti-inflammatory, antioxidative, and gut microbiota-regulating activities. Due to these multifunctional biological effects, polyphenols have attracted considerable interest as supportive therapeutic agents in IBD (Li et al. 2025; Pero et al. 2009). Evidence reported by Pero et al. (Jang et al. 2017) indicates that quinic acid, a phenolic constituent obtained from millet, stimulates intestinal metabolic pathways involved in the production of tryptophan and nicotinamide. This metabolic modulation contributes to enhanced genomic maintenance and to the suppression of NF-κB signaling, mediated by increased intracellular availability of these metabolites. In addition, quinic acid has been shown to exert protective effects against vascular inflammation and atherogenesis by interfering with MAPK- and NF-κB-dependent pathways and by limiting the expression of endothelial adhesion molecules (Ghasemi-Dehnoo et al. 2023). Consistently, an in vivo study demonstrated (Chassaing et al. 2017) that quinic acid administration markedly improved experimental UC in rats, accompanied by a significant reduction in NF-κB-related gene transcription.

Dietary interventions play a pivotal role in modulating the intestinal microbiota and constitute an essential element of IBD and CVD management. Accumulating evidence indicates that particular dietary constituents, including food emulsifiers, can directly influence microbial community structure and microbial gene expression, thereby exacerbating intestinal inflammatory responses (Chassaing et al. 2017; Vrdoljak et al. 2022). Among established nutritional strategies, dietary restriction, such as caloric limitation and fasting-mimicking regimens, has attracted considerable attention. These approaches have been shown to attenuate intestinal inflammation and promote epithelial regeneration in patients with IBD, largely by altering the production of metabolites and signaling molecules that regulate host metabolic pathways (Pavlović et al. 2025; Schmidt and Lorentz 2021; Ashton et al. 2019).

Exclusive enteral nutrition (EEN) represents another well-validated dietary intervention and involves the exclusive consumption of a nutritionally complete liquid formula. EEN has demonstrated substantial efficacy in inducing remission in pediatric CD, with reported response rates of up to 80% (Pigneur et al. 2019). Remission achieved through EEN is frequently accompanied by enhanced mucosal healing, which correlates with marked shifts in gut microbiota composition (Ormsby, et al. 2019). Furthermore, ethanolamine, a nutrient that supports the growth of several intestinal pathogens, including adherent-invasive *Escherichia coli* (AIEC), has been implicated in disease pathogenesis. Notably, EEN therapy has been shown to downregulate ethanolamine utilization operons in patients with CD, suggesting a microbiota-mediated mechanism contributing to its therapeutic effects (Miller et al. 2026).

FMT entails the transfer of intestinal microbial communities from healthy donors into the gastrointestinal tract of recipients with the objective of re-establishing microbial homeostasis and treating a range of gastrointestinal and systemic disorders (Suez et al. 2018). Through restoration of a balanced intestinal microbiota and its associated metabolic functions, FMT has emerged as a promising strategy for therapeutic modulation of the gut microbiome in IBD (Krynicka et al. 2026; Paramsothy et al. 2019). Clinical evidence indicates that FMT administration in patients with UC leads to significant increases in microbial richness and evenness, with measurable effects on both luminal and mucosa-associated microbial populations. Shotgun metagenomic analyses have further demonstrated that these microbiome alterations are sustained for up to eight weeks following treatment (Tan et al. 2022). However, considerable heterogeneity exists across studies regarding donor selection, stool preparation (fresh versus frozen), dosing regimens, route of administration, and patient populations, complicating definitive conclusions about efficacy and optimal protocols. Moreover, while most adverse events reported have been mild and self-limiting, occurrences of IBD flares and serious events such as hospitalization or disease exacerbation have been documented in subsets of patients, underscoring the need for cautious interpretation of safety data and standardized long-term surveillance. The cumulative evidence suggests that FMT is a promising but not fully standardized microbiome-based therapeutic strategy in IBD, warranting further large-scale, well-controlled trials to clarify its efficacy, safety, and long-term outcomes (Qazi et al. 2017; Doukas et al. 2025; Šantić et al. 2025; Noble et al. 2025).

Furthermore, it should be taken into account that current immunosuppressive therapy used in IBD could have opposite effects on cardiometabolic function, both beneficial in the case of aminosalicylates or anti-TNF-alpha and adverse in the case of corticosteroids, so that multidisciplinary approach should be provided for personalized treatment depending on the cardiovascular function of each subject (Wu et al. 2021).

At present, there are no IBD treatments specifically designed to target archaea; however, several promising approaches are beginning to emerge. One such strategy focuses on promoting beneficial archaeal populations. For instance, if reduced levels of *M. smithii* are characteristic of IBD, its supplementation may offer therapeutic value. In this context, probiotics based on methanogenic archaea could potentially increase SCFA production or alleviate bloating, an idea supported by earlier findings in ruminant studies (Li et al. 2024).

However, challenges remain due to cross-species differences in microbiota and inflammatory responses when IBD therapies are tested in animal models, posing translational challenges and highlighting the need for improved animal models and human studies.

**Fig. 2:**
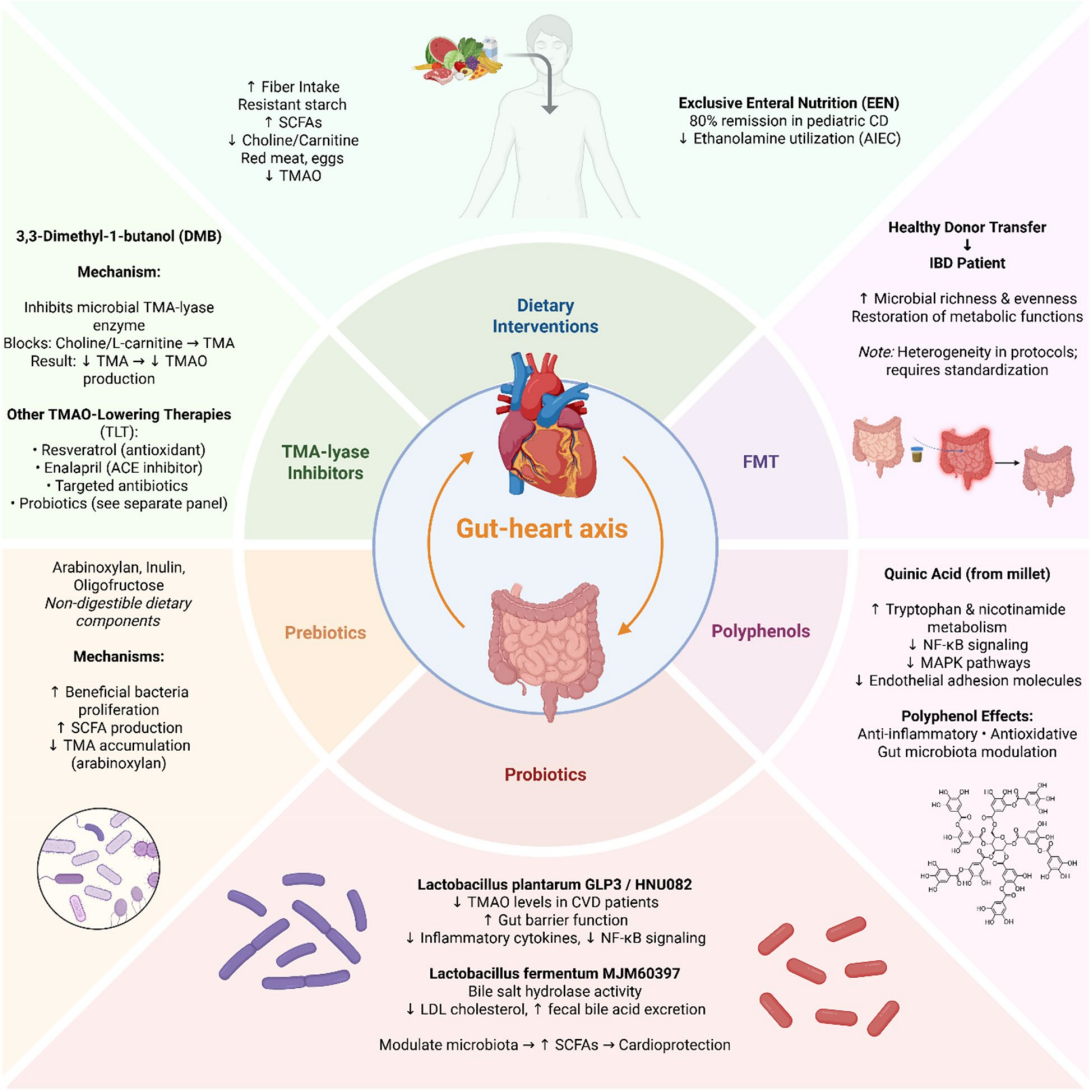
Therapeutic Modulation of the Gut-Heart Axis: Dietary and Microbiota-Targeted Interventions. The figure summarizes major therapeutic strategies targeting the gut-heart axis to reduce cardiovascular risk in patients with IBD and cardiometabolic comorbidities. Interventions include dietary modification, inhibition of TMA production, prebiotics, probiotics, polyphenols, and FMT. Dietary interventions emphasize increased intake of dietary fiber and resistant starch, leading to enhanced SCFA production, alongside reduced consumption of choline- and carnitine-rich foods (e.g., red meat and eggs), thereby lowering circulating TMAO levels. Microbial TMA-lyase inhibitors, such as DMB, block the conversion of dietary choline and L-carnitine to TMA, thereby reducing TMAO production. Additional TMAO-lowering approaches include polyphenolic compounds (e.g., resveratrol), angiotensin-converting enzyme (ACE) inhibitors (e.g., enalapril), targeted antibiotics, and selected probiotics. Prebiotics (e.g., arabinoxylan, inulin, oligofructose) promote beneficial bacterial proliferation, increase SCFA production, and reduce intestinal TMA accumulation. Probiotic strains such as Lactobacillus plantarum GLP3/HNU082 and Lactobacillus fermentum MJM60397 improve gut barrier integrity, reduce inflammatory cytokine production via inhibition of NF-κB signaling, modulate bile acid metabolism, lower LDL cholesterol, and decrease fecal bile acid reabsorption, collectively contributing to cardioprotection. Polyphenols (e.g., quinic acid derived from millet) modulate tryptophan and nicotinamide metabolism, suppress NF-κB and MAPK signaling pathways, and reduce endothelial adhesion molecule expression, exerting anti-inflammatory and antioxidative effects while reshaping gut microbial composition. FMT from healthy donors to IBD patients increases microbial richness and evenness and partially restores metabolic functions, although heterogeneity of protocols remains a limitation. Collectively, these strategies highlight the therapeutic potential of targeting gut microbiota and microbial metabolites to beneficially modulate the gut-heart axis and reduce cardiovascular disease risk. Created with BioRender

An overview of microbiota-targeted therapeutic strategies and their proposed effects on microbial metabolites and cardiometabolic risk in IBD is summarized in Fig. 2.

## Limitations of current knowledge and potential areas for future researh

Currently, routine cardiovascular screening has not been proposed in IBD-specific guidelines. Instead, risk factor assessment and management are individualized based on patient comorbidities. However, periods of active disease require increased monitoring for the development of CVD.

Microbiota-derived metabolites have emerged as pivotal links between intestinal inflammation and cardiovascular comorbidity in IBD. Proatherogenic metabolites promote endothelial dysfunction and vascular injury, whereas SCFAs and balanced bile acid signaling exert anti-inflammatory and cardioprotective effects, positioning the gut-heart axis as a compelling therapeutic target (Noble et al. 2025; Wu et al. 2021; Kumric et al. 2023; Kochkarian et al. 2025). Circulating levels of microbial metabolites such as TMAO, PAGln, IS, and specific bile acids may add incremental prognostic information for cardiovascular risk stratification in patients with IBD, potentially outperforming classical risk models when combined with markers of intestinal inflammation. Translating these mechanistic insights into clinical practice may enable personalized, microbiota-based strategies to mitigate cardiovascular risk in IBD.

There is currently a lack of specific insight into the predicted impact of IBD therapies—such as corticosteroids and biologics—on cardiovascular risk, particularly when stratified by disease phenotype (e.g., penetrating versus luminal CD). Moreover, the literature does not provide sufficiently detailed information on how disease duration, subtype, activity level, and phase-specific therapeutic interventions influence the gut microbiome in IBD patients.

In addition, stronger integration is needed between dietary patterns and microbiome-related cardiovascular risk. Specifically, the beneficial effects of the Mediterranean diet and the detrimental impact of a “cafeteria-style” diet should be more clearly linked to microbiome alterations in IBD that directly modulate cardiovascular risk (Arrari et al. 2024).

Taken together, these gaps underscore the need for a multidisciplinary approach to IBD management, incorporating not only disease control but also targeted nutritional education of IBD patients. Current therapeutic strategies remain insufficient in addressing specific cardiovascular risk pathways; for example, it is still unclear how IBD-associated dysbiosis independently contributes to conditions such as hypertension or non-hypertensive cardiovascular disease, and how prophylactic cardiovascular treatment strategies could be tailored according to IBD subtype (e.g., proctitis, left-sided colitis, pancolitis in ulcerative colitis, or ileal-dominant Crohn’s disease).

Although numerous review articles have explored the gut-heart axis, the novelty of this review lies in its exclusive focus on the IBD-heart axis, with particular emphasis on microbiome disruption in IBD patients. The key takeaway is that significant therapeutic challenges remain in leveraging microbiome modulation to reduce cardiovascular risk in IBD, and that well-designed clinical studies are urgently needed to address these gaps.

## Conclusions

Despite advances in defining mucosa-associated microbiota, progress remains constrained by methodological heterogeneity, predominantly observational study designs, and limited causal validation of host-microbe-metabolite interactions. Inconsistent sampling and sequencing approaches, insufficient longitudinal human cohorts, and challenges in resolving strain-level functional heterogeneity impede mechanistic clarity and clinical translation. Addressing these gaps will require standardized spatial-omics frameworks, functional validation platforms, and well-powered longitudinal studies to move the field from association to actionable insight, ultimately improving cardiovascular and intestinal outcomes in IBD.

## Data Availability

No datasets were generated or analysed during the current study.
